# Monitoring adherence to antiretroviral therapy among adolescents in Southern Uganda: comparing Wisepill to Self‐report in predicting viral suppression in a cluster‐randomized trial

**DOI:** 10.1002/jia2.25990

**Published:** 2022-09-02

**Authors:** Samuel Kizito, Flavia Namuwonge, Rachel Brathwaite, Torsten B. Neilands, Proscovia Nabunya, Ozge Sensoy Bahar, Christopher Damulira, Abel Mwebembezi, Claude Mellins, Mary M. McKay, Fred M. Ssewamala

**Affiliations:** ^1^ International Center for Child Health and Development, Brown School Washington University in St. Louis St. Louis Missouri USA; ^2^ International Center for Child Health and Development Masaka Uganda; ^3^ Division of Prevention Science University of California San Francisco California USA; ^4^ Reach the Youth Uganda Kampala Uganda; ^5^ Columbia University, HIV Center for Clinical and Behavioral Studies Department of Psychiatry New York State Psychiatric Institute and Columbia University New York City New York USA

**Keywords:** ART adherence, economic empowerment, real‐time monitoring, HIV/AIDS, adolescents, sub‐Saharan Africa

## Abstract

**Introduction:**

Optimal antiretroviral therapy (ART) adherence is crucial for improved patient outcomes; however, ART adherence among adolescents living with HIV (ALHIV) is low. Also, the performance of various adherence measures among ALHIV is under contention. We monitored ART adherence and compared Self‐report (SR) and Wisepill electronic monitoring (EM) performance in measuring ART adherence and predicting HIV viral suppression among ALHIV.

**Methods:**

Between January 2014 and December 2015, we recruited 702 ALHIV aged 10–16 years into our cluster‐randomized controlled trial (2012–2018) in 39 clinics in Uganda. The intervention included a long‐term savings child development account, four micro‐enterprise workshops and 12 mentorship sessions. Using the entire sample, we performed multilevel logistic regression to predict monthly ART adherence trends for the first year of follow‐up. Since it is possible that the intervention had different effects on SR and EM adherence, we used participants in the control arm only to compare adherence using SR and EM and to calculate their sensitivity and specificity in predicting viral suppression.

**Results:**

There was a significant decline in adherence for each month throughout the entire follow‐up period regardless of the group assigned. Good ART adherence was measured at 79.2% (75.2–82.6%) and 97.0% (95.4–98.1%) using EM and SR, respectively. Overall, 64.3% (60.6–67.9%) had suppressed viral loads. The specificities for EM and SR in predicting viral non‐suppression were 80.4% (73.6–85.7%) and 96.7% (93.3–98.4%), while the sensitivities were 22.9% (15.0–33.3%) and 1.8% (0.4–6.9%), respectively. The area under the curve was low for both EM and SR, at 53.6% (45.7–61.5%) and 56.2% (53.2–59.3%), respectively. There was high agreement (78%) between SR and EM in monitoring adherence.

**Conclusions:**

Our findings highlighted the need for strategies for sustained optimal adherence. SR and EM measure adherence with a considerable agreement; however, neither is an accurate predictor of virological outcome. There is still a need for an acceptable, feasible and affordable method that predicts viral suppression among ALHIV.

## 1 INTRODUCTION

Most children born with HIV survive into adolescence and adulthood due to expanded access to antiretroviral therapy (ART) [1, 2]. This, alongside horizontal transmission, has resulted in the number of adolescents living with HIV (ALHIV) to increase in the last decade. Approximately 1.5 million ALHIV reside in sub‐Saharan Africa, accounting for 88% of the global population of ALHIV [3, 4, 7]. In Uganda, of the 1.2 million people living with HIV, 150,000 are children and adolescents below 15 years [4].

Optimal ART adherence is associated with better clinical and immunological outcomes, such as preventing HIV drug resistance, reducing HIV transmission and prolonging survival. Compared to adults, ALHIV have lower ART adherence and poorer outcomes [3, 5, 6, 28, 38], resulting in increased AIDS‐related deaths in this population [7]. In 2019 alone, globally, 34,000 adolescents died from AIDS and AIDS‐related causes [7]. In Uganda, viral suppression among ALHIV is 32.5% and 44.9% for males and females, respectively [5]. Gaps still exist in research on adherence in this group. For instance, although electronic monitoring (EM) allows objective measurement of ART adherence prospectively, few studies employing EM have focused exclusively on adolescents from low‐income backgrounds [8]. Yet, understanding the trend in adherence is crucial in informing the development of focused interventions to improve adherence.

Self‐reports (SR) and EM are two of the various measures of ART adherence [3, 9, 10, 11, 29, 30, 39, 40]. Although these measures perform well in adults [11, 12], their performance in ALHIV is variable. For example, in young adolescents, the responsibility of ensuring adherence lies on both the ALHIV and the caregiver [13, 14, 41, 42]. Therefore, using SR to study adherence in this age group requires consideration of both the ALHIV and the caretaker. However, SR is often administered to the adolescent alone, without considering the caretaker. Also, because adolescents are still developing cognitively, their reporting behaviours may differ from those of adults [13]. SR is subject to recall and social desirability biases leading to over‐estimation of adherence [3, 15]. EM measures are costly, and usually require reliable telephone network connectivity [3, 15]. There is a need to understand how these measures perform in monitoring adherence and predicting viral suppression in resource‐limited settings. Our paper had two main aims: (1) to model the monthly changes in the prevalence of EM adherence to ART and (2) to compare the performance of SR and EM ART adherence in predicting HIV viral suppression among ALHIV in a resource‐limited setting.

## 2 METHODS

### 2.1 Study design

This paper utilized data from a 5‐year NICHD‐funded cluster‐randomized controlled trial (Suubi+Adherence study) that ran between 2012 and 2018. The study examined the impact of a family‐based economic empowerment intervention on HIV treatment adherence among ALHIV. The intervention package had three components including: (1) a child development account for long‐term saving, (2) four microenterprise workshops training participants and their families on how to save money and start a family business, and (3) 12 mentorship sessions addressing financial planning, business development and setting up short‐ and long‐term goals. The workshops were provided to the participants and their families, while the mentorship sessions were offered for the adolescents only. Adherence assessments were conducted at baseline, 12‐, 24‐, 36‐ and 48‐months follow‐ups. The details have been published in the study protocol and other International Center for Child Health and Development publications [16, 17, 43, 44].

### 2.2 Study setting

Participants were recruited from health clinics located in the Greater Masaka region in Uganda. The area reports HIV prevalence rates of up to 12%, which is higher than the national prevalence [18]. Clinics were included in the study if they were accredited to provide HIV care.

### 2.3 Study population

Between January 2014 and December 2015, we recruited 702 HIV‐positive adolescents (control = 344 and intervention = 358) and followed them up for 4 years. Briefly, to be eligible for enrolment into the study, one had to be HIV positive (adolescent was tested and had confirmation from the medical report) and aware of their status, between ages 10 and 16 years, living within a family and on ART. These patients visited the clinic monthly as part of the routine clinic procedures. When modelling the monthly changes in ART adherence, we included all the participants irrespective of the study group. On the other hand, to compare the performance of SR and EM in predicting HIV viral suppression, we included only participants in the control group.

### 2.4 Randomization

**Figure 1:**
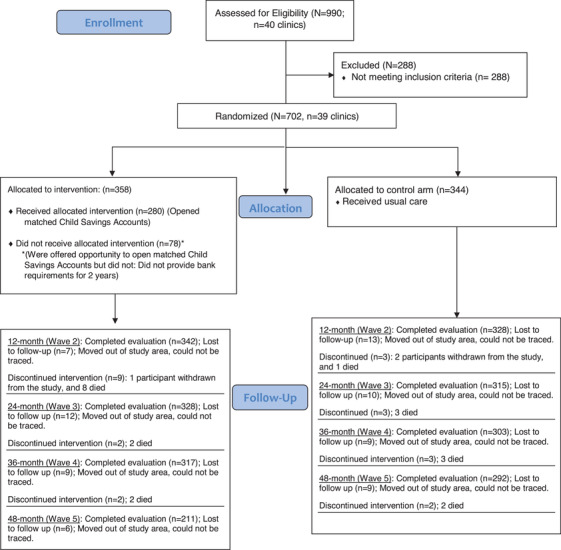
CONSORT flow chart for the Suubi+Adherence study (2012–2018). In the current study, we followed up the participants for the first year of the study. To answer the first aim of our current study, we included participants from both study groups. For the second study aim, we only included participants in the control group and used measurements taken at 1 year of follow‐up to compare the performance of SR and EM in monitoring adherence and predicting viral suppression. In the intervention group, 342 of the 356 participants completed evaluation at the end of the first year. Of the 16 participants who did not complete the evaluation, seven were lost to follow‐up, one participant withdrew from the study and eight participants died. In the control group, 328 of the 344 participants completed evaluation at the end of the first year. The 16 participants who did not complete the evaluation included 13 participants who were lost to follow‐up, two participants who withdrew from the study and one participant who died.

Initially, 40 clinics were randomized into the two study arms using the restricted randomization technique. However, before data collection started, one clinic was closed by the district health officials because it lacked proper operational licensure. This clinic was subsequently dropped from the study leaving 39 clinics (19 clinics in the control arm and 20 clinics in the intervention arm) (Figure 1). All the participants in a clinic received the same intervention determined by the study arm in which the clinic was randomized.

## 3 MEASURES

### 3.1 Adherence measures

#### 3.1.1 Electronic adherence monitoring

Participants were provided with a Wisepill adherence monitoring device [19, 20] connected to a mobile telecommunications network. Whenever a patient opened the device, it sent a signal to a central server. This signal was denoted as “intake” and was a proxy for a dose taken. Each time the device was not opened, it registered a signal denoted “heartbeat,” which indicated that the device was working, but it was not opened. A signal denoted “none” was only sent when the device was malfunctioning. We received daily adherence information from the participants’ Wisepill device. A missed dose was coded with 0, and a dose taken was coded as 1. We aggregated the daily adherence data to generate monthly adherence. Using the 2020 Consolidated Guidelines for the Prevention and Treatment of HIV in Uganda [21], we dichotomized adherence into good and poor. The guidelines define poor ART adherence as missing ART for more than 4 days a month. At analysis, the few times the devices were malfunctioning were treated as missing data. Patients opened their Wisepill device several times a day for the first few days of receiving the device. As a result, the devices transmitted numerous signals every day for most of the first week. Therefore, due to concerns regarding the reliability of the device opening data during the initial usage period, we excluded the data from the first week after receiving the Wisepill device.  After the first week, for instances where the participants opened the Wisepill device more than once for a prescribed dose, we considered only the first opening and ignored the subsequent openings.

#### 3.1.2 Self‐reported ART adherence

Each participant was asked to recall how many days they had missed taking at least one of the doses of their HIV medication in the preceding 30 days. Specifically, the participant was asked, “*In the last 30 days, on how many days did you miss at least one dose of your HIV medications?*” This measure has been previously used [16, 17]. We dichotomized the adherence into good and poor adherence based on missing at least one dose of ART for more than 4 days in a month [21].

#### 3.1.3 Viral load

The viral loads were quantified using the Abbott RealTime HIV‐1 RNA PCR, version 5.00. The viral load was dichotomized into suppressed (<50 copies per ml) and unsuppressed (≥50 copies/ml) [22].

#### 3.1.4 Baseline characteristics

We collected information about socio‐demographic characteristics, including, sex (males vs. females) and orphanhood status (not orphan vs. single orphan vs. double orphan). We also collected treatment‐related information, including the antiretrovirals (ARV) treatment regimen (first vs. second vs. third line), the number of pills taken per day (less than two vs. two to four vs. more than four) and the frequency of taking the ART in a day (once vs. twice).

### 3.2 Data analysis

Data were analysed using Stata version 15.1. We summarized numerical data using means and standard deviations, and categorical data using percentages. We compared baseline socio‐demographic and treatment characteristics between the intervention and control arm (Table 1). Depending on the distribution, we used survey estimator analogs of the *t*‐test or a Mann–Whitney‐U test for the numerical data and the Rao–Scott adjusted chi‐square test for categorical data. Of the 702 participants, 103 did not receive a Wisepill device and were not included in the analysis for aim one (modelling the ART adherence trends) Seventy‐eight of the participants without a Wisepill device were randomized to the control group. We performed subgroup analysis based on age, sex, orphanhood status, ARV regimen, number of pills prescribed and frequency of taking ART. We also performed a sensitivity analysis for various SR, EM and viral load cut‐off values.

**Table 1:** Baseline characteristics of 702 adolescents living with HIV in Uganda

Characteristics	Total sample *n* = 702 (%)	Control *n* = 344 (%)	Intervention *n* = 358 (%)	
Female	339 (56.3)	174 (50.1)	165 (64.0)	0.783
Age in completed years (mean ± SD)	12.4 ± 1.98	12.4 ± 1.97	12.5 ± 1.99	0.493
Orphanhood status^a^				
Both parents are deceased	182 (26.4)	95 (28.0)	87 (24.8)	0.537
One parent is still alive	262 (38.0)	129 (38.1)	133 (37.9)	
Both parents are alive	246 (35.7)	115 (33.9)	131 (37.3)	
ARV treatment regimen				
First‐line treatment regimen	438 (62.4)	222 (64.5)	216 (60.3)	0.251
Second‐line treatment regimen	258 (36.8)	117 (34.0)	141 (39.4)	
Third‐line treatment regimen	6 (0.9)	5 (1.5)	1 (0.3)	
Number of pills prescribed per day				
Less than 2	445 (63.4)	218 (63.4)	227 (63.4)	0.967
2–4	155 (22.1)	77 (22.4)	78 (21.8)	
More than 4 pills	102 (14.5)	49 (14.2)	53 (14.8)	
Baseline viral load				
Suppressed (<50 copies/ml)	424 (60.4)	219 (63.7)	205 (57.3)	0.083
Not suppressed (≥50 copies/ml)	278 (39.6)	125 (36.3)	153 (42.7)	
Frequency of medication				
Once a day	75 (12.7)	36 (12.4)	39 (13.0)	0.622
More than once a day	515 (87.3)	254 (87.6)	261 (87.0)	

Abbreviation: SD, standard deviation.

^a^ Some participants (12) had missing information on orphanhood status.

#### 3.2.1 Multilevel regression analysis

To longitudinally explore the effect of the intervention on EM ART adherence, we performed multilevel logistic regression models using the *melogit* command. We estimated the margins (i.e. predicted probabilities) for our model and then generated a margins plot for the predicted adherence against time. After ruling out group‐time interaction through running contrasts of marginal predictions, we ran further contrast commands to determine whether the change in adherence over time was significant. Statistical significance for all effects was evaluated at alpha = 0.05.

#### 3.2.2 Agreement between measures of adherence

In comparing the performance of the SR and EM adherence in predicting viral suppression, we included only participants in the control arm of the parent study. We excluded participants in the intervention arm to ensure that any observed differences in adherence were not attributed to the intervention. We used viral load, SR and EM adherence data collected during the 12th month of follow‐up. This way, we ensured that we compared results for measures collected at the same time point. We calculated the percentage agreement between the three tests and the Kappa statistic to determine whether the observed percentage agreement was not due to chance. As observed in our data, whenever there is skewness in the data compared, Kappa statistic is prone to the “paradox of kappa,” whereby the kappa values are so low despite high observed agreement [23]. We employed the adjusted coefficient (AC_1_) proposed by Gwet in 2008, which adjusts for the “paradox of Kappa” bias [24].

#### 3.2.3 Area under the curve

We determined the sensitivity and specificity of SR and EM adherence in predicting viral non‐suppression; we fitted a model to generate the area under the curve (AUC) and associated bootstrapped 95% confidence intervals using the cluster bootstrap to control for clustering at the clinic level. We included a subgroup analysis among young (below 14 years) and older (14–16 years) adolescents. AUC below 70% shows the test is poor, while values of 70–80%, 80–90% and above 90% represent acceptable, excellent and outstanding test performances, respectively [25]. We plotted marginal receiver‐operator curve (ROC) curves for SR and EM adherence in predicting viral outcomes.

### 3.3 Ethical considerations

The study was approved by the Makerere University School of Public Health Research and Ethics committee (Protocol #210) and the Uganda National Council for Science and Technology (UNCST, SS 2969). Also, the study received approval from the Columbia University Review Board (AAAK3852). Suubi+Adherence study was registered at ClinicalTrials.gov (#NCT01790373). Adolescents provided informed written assent, and caregivers provided written consent before participating in the study. All study staff received research ethics training in Good Clinical Practice or online Collaborative Institutional Training Initiative.

## 4 RESULTS

### 4.1 Socio‐demographic characteristics

Table 1 presents the baseline socio‐demographic characteristics of the study population. These characteristics were comparable between the control and intervention arms. Overall, the sample comprised 56.3% females (*n* = 339), and the mean age was 12.4 years. Regarding orphanhood status, 38.0% (*n* = 262) had lost one parent, while 35.7% (*n* = 246) had both parents alive. At baseline, 60.4% (*n* = 424) had suppressed viral loads. There were no significant differences in the distribution of baseline characteristics based on viral load suppression (Table 2).

**Table 2:** Distribution of participant characteristics based on viral suppression

Viral load; *N* (percentage)			
Characteristics	Suppressed	Unsuppressed	*p* value
Female	245 (57.8)	151 (54.3)	0.365
Age in completed years			
10–13 years	294 (69.4)	183 (65.8)	0.329
14–16 years	130 (30.7)	95 (34.2)	
Orphanhood status			
Both parents are deceased	104 (24.9)	78 (28.7)	0.420
One parent is still alive	158 (37.8)	104 (38.2)	
Both parents are alive	156 (37.2)	90 (33.1)	
ART regimen			
First‐line treatment regimen	275 (64.9)	163 (58.6)	0.096
Second and third regimen	149 (35.1)	115 (41.4)	
Frequency of medication			
Once a day	46 (12.3)	31 (13.5)	0.667
More than once a day	327 (87.7)	198 (86.5)	

Abbreviation: ART, antiretroviral therapy.

### 4.2 ART adherence

**Figure 2:**
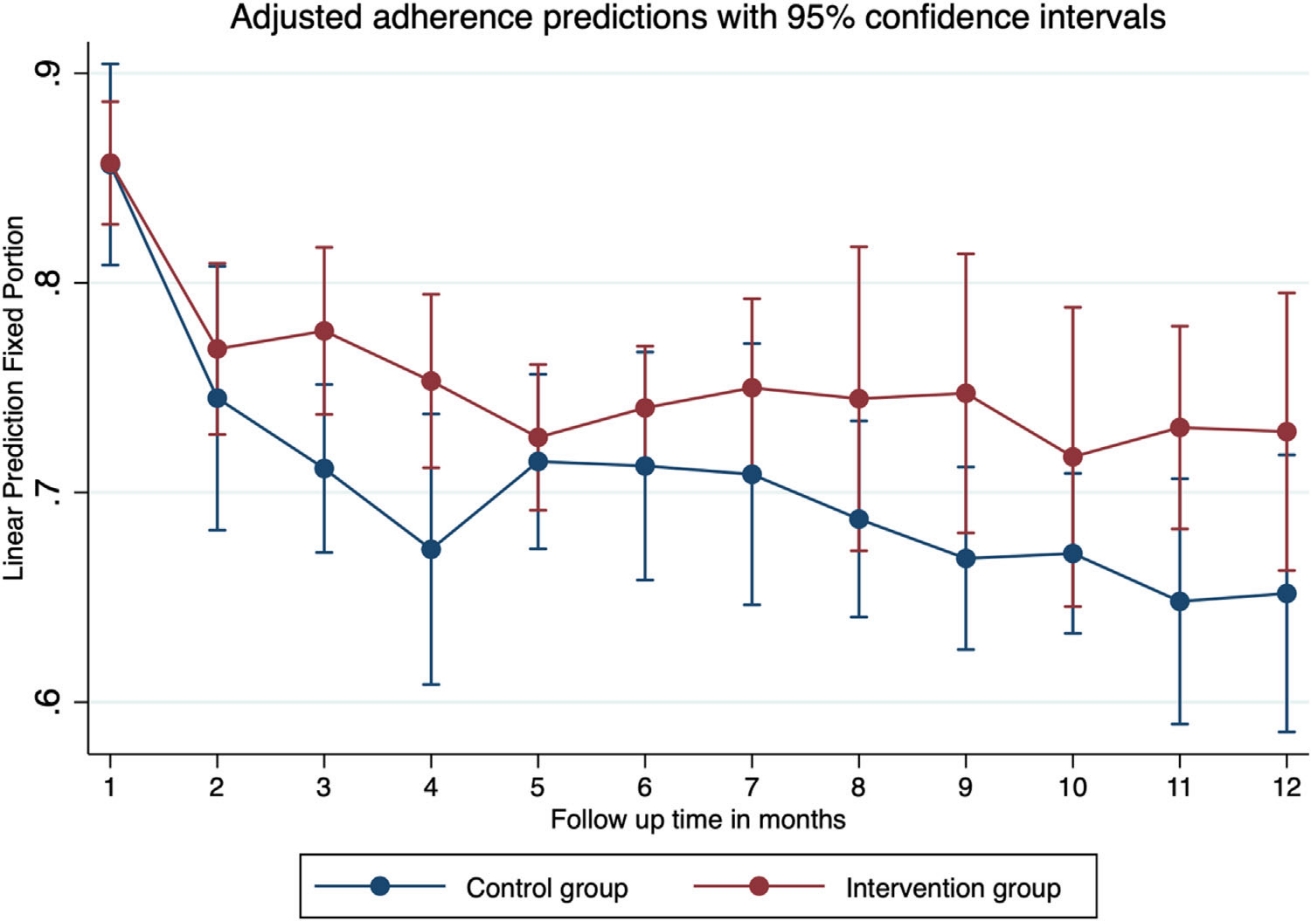
Linear prediction of monthly adherence measured using the Wisepill device in the Suubi+Adherence study conducted among ALHIV in Southern Uganda. We fitted a three‐level logistic regression model, with the repeated measures of adherence taking level 1, the participant at level 2 and the clinic at level 3. Vertical bars denote the 95% confidence intervals (CI) for the adherence in each of the months of follow‐up. Optimal adherence meant that a participant took their ART and missed medication on only 4 days or less within the month. A total of 599 ALHIV contributed to 6262 observations over a period of 1 year. EM data were generated daily and were averaged to generate monthly adherences.

The multi‐level regression model for predicting ART adherence measured using the Wisepill device showed that there was no significant difference in adherence between the intervention and control arm β = 0.339 (95% CI: –1.094 to 1.771), *p* = 0.643. Also, compared to the first month, there was a significant decline in adherence for each month throughout the entire follow‐up period regardless of the group assigned. Compared to baseline, there was significantly lower adherence at each follow‐up point, irrespective of the study group (Table 3). The findings are illustrated in Figure 2. The monthly adherence gradually declined for the first 5 months of follow‐up, after which it showed monthly fluctuations for the rest of the follow‐up time.

**Table 3:** Monthly trends in ART adherence among adolescents with HIV measured using Wisepill technology for 1 year among ALHIV

		95% Confidence interval		
Variable	Coefficient	Lower limit	Upper limit	*p* value
Time in months				
1	Reference			
2	−1.202	−2.090	−0.313	0.008
3	−1.787	−2.906	−0.668	0.002
4	−1.875	−2.808	−0.943	<0.001
5	−2.177	−3.051	−1.304	<0.001
6	−2.217	−3.091	−1.343	<0.001
7	−2.166	−2.930	−1.402	<0.001
8	−2.473	−3.450	−1.496	<0.001
9	−2.373	−3.589	−1.158	<0.001
10	−2.517	−3.797	−1.236	<0.001
11	−1.798	−3.284	−0.670	0.003
12	−2.416	−3.717	−1.115	<0.001
Group				
Intervention	0.339	−1.094	1.771	0.643
Time and group interaction				
1	Reference			
2	−0.415	−1.639	0.808	0.506
3	−0.217	−1.614	1.181	0.761
4	−0.091	−1.329	1.147	0.885
5	−0.369	−1.726	0.987	0.594
6	−0.069	−1.434	1.296	0.921
7	0.573	−0.823	1.968	0.421
8	0.021	−1.476	1.518	0.978
9	0.190	−1.415	1.794	0.817
10	−0.176	−1.819	1.468	0.834
11	−0.343	−1.931	1.245	0.672
12	−0.116	−1.921	1.689	0.900
Constant	6.044	4.778	7.311	<0.001
**Random effects**	**Coefficient**	**Lower limit**	**Upper limit**	
Clinic variance	0.089	0.014	0.528	
Participant variance	10.610	8.208	13.716	

### 4.3 Agreement between the adherence measures

Using EM, 79.2% (75.2–82.6%) of the participants had good ART adherence, while 97.0% (95.4–98.1%) reported good adherence with SR. Overall, 64.3% (60.6–67.9%) had suppressed viral loads at the 12th month of follow‐up. We observed statistically significant differences in viral suppression across participants who were prescribed 2 or fewer pills, 2–4 and more than 4 pills per day (*p* = 0.022). Also, the self‐reported adherence was significantly higher in males than females (*p* = 0.05). See Table 4.

**Table 4:** Adherence to antiretroviral therapy measured at month 12 of follow‐up among adolescents with HIV

	Viral suppression^a^		Wisepill adherence^b^		Self‐reported adherence^b^	
Variables	Prevalence (95% CI)	*p* value	Adherence	*p* value	Adherence	*p* value
Overall	64.3 (60.6–67.9)		79.2 (75.2–82.6)		97.0 (95.4–98.1)	
Age of adolescent						
10–13 years	64.9 (60.5–69.2)	0.625	78.1 (73.0–82.4)	0.425	96.8 (94.7–98.0)	0.553
14–16 years	63.0 (56.2–69.3)		81.8 (74.5–86.5)		97.6 (94.3–99.0)	
Sex						
Male	62.4 (56.7–67.7)	0.349	80.8 (74.7–85.7)	0.454	95.6 (92.5–97.4)	0.050
Female	65.9 (60.9–70.5)		77.9 (72.5–82.6)		98.1 (96.1–99.1)	
Orphanhood						
Double orphan	62.9 (30.1–44.6)	0.704	82.7 (74.8–88.5)	0.407	97.6 (93.9–99.1)	0.708
One parent alive	63.3 (57.1–69.1)		79.4 (72.9–84.8)		96.8 (93.7–98.4)	
Not orphan	66.4 (60.1–72.1)		76.1 (68.8–82.1)		97.9 (95.1–99.1)	
ART regimen						
First line	65.1 (60.4–69.6)	0.265	80.6 (75.6–84.8)	0.336	97.8 (95.8–98.9)	0.131
Second or third line	63.8 (57.7–69.5)		76.9 (70.2–82.5)		95.8 (92.5–97.6)	
Pills prescribed per day						
Less than 2	65.5 (60.8–69.9)	0.022	78.5 (73.4–82.8)	0.514	97.8 (95.9–98.9)	0.167
2–4	68.8 (61.0–75.7)		77.7 (68.5–84.8)		94.8 (89.9–97.4)	
More than 4 pills	52.5 (42.6–62.2)		84.3 (73.6–91.2)		97.0 (90.9–99.0)	
Frequency of medication						
Once a day	69.4 (57.7–79.1)	0.556	82.0 (70.0–89.8)	0.566	98.6 (90.5–99.8)	0.441
Twice a day	65.9 (61.7–70.0)		78.8 (74.5–82.5)		97.0 (95.1–98.2)	

Abbreviation: ART, antiretroviral therapy.

^a^ Viral suppression was defined by having a viral load of less than 50 copies/ml.

^b^ Good adherence was defined as a patient missing ART on only 4 days or less with in the last 30 days.

There was 77.7% agreement between SR and EM when monitoring adherence. However, the observed agreement was lower between SR versus viral load and EM versus viral load (64.0% and 61.4%, respectively). The kappa statistic for all three tests was low, ranging from 0.02 to 0.06. However, the agreement coefficients were higher, ranging from 0.4 to 0.8, which suggested moderate to good agreement between the measures. Comparable results were observed in younger (10–13 years) and older (14–16 years) adolescents (Table 5).

**Table 5:** Agreement between the measures of ART adherence among ALHIV in the control group

Adherence measure^a^	Observed agreement	Expected agreement	Adjusted expected agreement	Kappa statistic	Agreement coefficient
Overall					
Viral load versus Wise pill	61.4%	59.9%	39.3%	0.04	0.364
Viral load versus Self‐report	64.0%	64.7%	30.8%	0.02	0.484
Wise pill versus Self‐report	77.7%	77.4%	21.0%	0.01	0.716
Among ALHIV aged 10–13 years					
Viral load versus Wise pill	61.1%	60.3%	39.5%	0.02	0.356
Viral load versus Self‐report	66.1%	66.9%	28.7%	0.02	0.590
Wise pill versus Self‐report	75.6%	74.9%	23.2%	0.03	0.682
Among ALHIV aged 14–16 years					
Viral load versus Wise pill	62.0%	58.4%	39.0%	0.09	0.792
Viral load versus Self‐report	59.0%	59.4%	33.8%	0.01	0.379
Wise pill versus Self‐report	82.3%	83.1%	16.2%	0.05	0.789

Abbreviation: ALHIV, adolescents living with HIV.

^a^ Viral load was fixed at 50 copies/ml cut‐off.

### 4.4 Sensitivity, specificity and AUC

**Figure 3:**
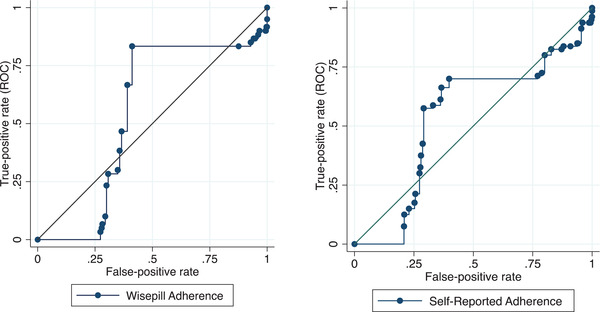
Area under the receiver‐operator characteristic curve for the prediction of virologic failure using Wisepill (left) and Self‐report (Right) at month 12 of follow‐up. Viral suppression was defined as having a viral load of at least 50 copies per ml. The marginal ROC curves were plotted after running a regression model to estimate the area under the curve. A total of 251 ALHIV contributed data for the EM adherence ROC (left), while 328 ALHIV contributed the data for the SR ROC graph.

We found high specificities of, 80.4% (73.6–85.7%) and 96.7% (93.3–98.4%), for the EM and SR, respectively. The sensitivities for the two tests were 22.9% (15.0–33.3%) and 1.8% (0.4–6.9%), respectively. The AUCs for EM and SR were 53.6% (45.7–61.5%) and 56.2% (53.2–59.3%), respectively. We observed similar results when we performed separate tests for the adolescents aged 10–13 years and those 14–16 years old. Figure 3 shows the ROC for SR and EM in predicting viral non‐suppression (Table 6).

**Table 6:** The sensitivity and specificity measures of Wisepill and Self‐report methods in predicting viral non‐suppression among ALHIV in the control group

	Viral load				
Categories	Suppressed	Unsuppressed	Specificity	Sensitivity	AUC (%)
Overall					
*Wisepill measure*					
Poor adherence	33 (63.5)	19 (36.5)			
Good adherence	135 (67.8)	64 (32.2)	80.4 (73.6–85.7)	22.9 (15.0–33.3)	53.6 (45.7–61.5)
*Self‐report measure*					
Poor adherence	7 (77.8)	2 (22.2)			
Good adherence	208 (65.2)	111 (34.8)	96.7 (93.3–98.4)	1.8 (0.4–6.9)	56.2 (53.2–59.3)
Age 10–13 years					
*Wisepill*					
Poor adherence	27 (67.5)	13 (32.5)			
Good adherence	92 (69.7)	40 (30.3)	77.3 (68.8–84.0)	24.5 (14.6–38.1)	54.0 (45.0–63.0)
*Self‐report measure*					
Poor adherence	5 (83.3)	1 (16.7)			
Good adherence	153 (67.4)	74 (32.6)	96.8 (92.6–98.7)	1.3 (0.2–9.1)	56.4 (53.6–59.2)
Age 14–16 years					
*Wisepill measure*					
Poor adherence	6 (50.0)	6 (50.0)			
Good adherence	43 (64.2)	24 (35.8)	87.8 (74.9–94.5)	20.0 (8.9–38.9)	51.6 (39.7–63.6)
*Self‐report measure*					
Poor adherence	2 (66.7)	1 (33.3)			
Good adherence	55 (59.8)	37 (40.2)	96.5 (86.7–99.1)	2.6 (0.3–17.4)	54.9 (50.0–59.7)

### 4.5 Sensitivity analysis

We conducted sensitivity analysis by performing comparisons of SR and EM using various adherence levels. Specifically, we used percentage adherences of 95%, 90% and 85% for each measure. We also performed analyses at viral load cut‐off values of <1000 copies/ml to denote viral suppression, as defined by the Uganda national HIV guidelines [21]. All the sensitivity analyses yielded comparable results.

## 5 DISCUSSION

In the context of this randomized controlled trial of ALHIV, we modelled the monthly changes in the EM adherence to ART for 1 year and compared the performance of SR and EM in predicting HIV viral suppression among ALHIV in Southern Uganda. ART adherence gradually declined, with monthly fluctuations, starting in the first month until the last month, and the intervention was not efficacious in improving ART adherence. We also found that only two‐thirds of the participants had achieved viral suppression. Adherence was independent of demographic and treatment‐related factors. While both measures roughly correlated with each other, neither SR nor EM was found to reliably predict viral suppression in this population. Our results add to the understanding of the discrepancy in measuring ART adherence and predicting viral suppression in ALHIV.

The gradual decline in EM adherence is consistent with previous follow‐up studies among youths [26, 27]. One possible explanation for the declining adherence is a loss of interest in using the Wisepill device. Some participants reported to our research assistants that they were not using the Wisepill devices consistently since the devices needed to be charged, while others were afraid to misplace the device. Although not assessed in our study, qualitative studies elsewhere have examined the reasons for the decline in using the Wisepill device. One study highlighted that patients complained about the Wisepill device being conspicuous and bulky [27]. In these instances, patients resorted to keeping their pills elsewhere and continued swallowing them without using the Wisepill device. The declining adherence that we found in our study highlights the need to implement strategies to ensure sustained adherence.

The high levels of SR and EM adherence among participants in this study were comparable to those reported in studies from similar settings [28, 29, 30, 31]. Because most participants in our study were ART experienced, resilience is a plausible explanation for the high levels of ART adherence. Also, the ALHIV were recruited from HIV clinics, where they receive comprehensive HIV care, including adherence counselling and monitoring. In 2015, Nabukeera‐Barungi et al. found adherence of 87% among a hospital‐based cohort of ALHIV in Uganda, comparable to our findings [32].

As found elsewhere, SR reported a higher adherence than EM [3, 9, 33, 34, 45, 46]. SR overestimates adherence, possibly due to social desirability bias and recall biases [15, 27]. Both SR and EM had high specificities, low sensitivities and small AUCs. The high specificity with low sensitivity findings is comparable with a study among young adults in China [35]. The high specificity suggests that high SR and EM adherences were good predictors of viral suppression, while low sensitivities meant that both SR and EM performed poorly in predicting participants with unsuppressed viral loads. One explanation for the poor prediction of viral non‐suppression is that other mechanisms could be responsible for viral non‐suppression, such as drug resistance, drug interactions and drug absorption and metabolism problems [36, 37]. The low AUCs suggest that both SE and EM are less reliable predictors of viral outcomes among ALHIV. Patient‐level factors, such as age, did not influence SR and EM performance. Although SR is prone to several biases, we have demonstrated that its performance is comparable to that of EM [27]. Our results imply that in settings with limited access to electronic adherence measures, SR can be used to monitor adherence among ALHIV with comparable accuracy.

SR and EM agreed 78% of the time while categorizing participants (adherent or non‐adherent) and only disagreed 22% of the time. The kappa statistic was low, at 0.01, implying that this agreement could be by chance. The low kappa (despite a high level of agreement between SR and EM) was explained by the skewed nature of adherence as measured by both SR and EM, whereby most participants reported good adherence [23]. When we adjusted for this bias, we observed a higher agreement coefficient of 0.72, which suggested good agreement between the two measures. There was a moderate agreement between viral load and each of the two adherence measures, SR and EM. Due to the variation in adherence rates during the follow‐up time, it is possible that the observed agreement between the adherence measures and the predication of viral suppression was affected by the time point that we used in making the inferential analyses. Consistent with other studies, in our study, a considerable number of the patients with non‐suppressed viral loads had good adherence and vice versa [6]. The results further emphasize the caution that, if possible, clinicians should not rely on adherence measures to predict viral suppression. When employed in isolation (without other interventions like reminder mobile short messages), the EM does not provide additional benefit in monitoring treatment adherence and predicting viral suppression.

### 5.1 Strengths and limitations

Our study followed a large cohort of participants using the Wisepill device for 1 year, which is longer than the follow‐up time for many studies that employed similar technology in ALHIV. Secondly, the study is among a handful of studies that prospectively collected information on various adherence measures in the same cohort of ALHIV concurrently. Hence, we were able to compare the performance of the different adherence measures. Finally, we employed robust statistical methods, including multi‐level logistic regression, to model adherence trends over time. Thus, we dealt with clustering and accounted for the effect of time and the intervention on adherence.

However, our study has some limitations. It is possible that some participants took their ART without using the Wisepill device, resulting in misclassification bias in adherence. We truncated data from the first week of measuring adherence using the Wisepill device. For some participants, there were multiple signals generated during this period. Excluding data from the first week of monitoring could introduce bias in our study. We experienced several technical challenges, such as interruptions in signal transmission and failure to send a signal due to a drained battery, which resulted in missing data in measuring adherence. To minimize the missed data, our field team followed up through phone calls or physical visits whenever there was a missed signal for more than 3 days. Also, we did not have data on ART resistance and duration of medication, which could have influenced viral suppression. Finally, the results should be generalized cautiously, especially among populations in urban and low HIV burden settings. This is because the study recruited adolescents from HIV care clinics in Makasa subregion, a rural area with an HIV prevalence higher than the national average. In these high HIV burden settings, ALHIV receive comprehensive ART adherence counselling and monitoring from the HIV clinics.

## 6 CONCLUSIONS

Our findings highlighted the need for strategies to ensure sustained optimal adherence over time. SR and EM measured adherence with a considerable agreement; however, neither was an accurate predictor of virological outcome. There is still a need for an acceptable, feasible and affordable method that predicts viral suppression among ALHIV.

## COMPETING INTERESTS

All authors declare no competing interests.

## AUTHORS’ CONTRIBUTIONS

FMS, CM and MMM were responsible for fund acquisition. SK, RB, PN, OSB, CM and FMS conceptualized the study. SK, RB, TBN, PN and FMS were responsible for the methodology. CD, OSB, PN and FMS oversaw and supervised data collection. SK, RB, TBN and CD conducted the data analysis. FMS and TN directly accessed and verified the underlying data reported in the manuscript. FN, PN, OSB, AM and FMS were responsible for the study administration and supervision. SK wrote the original draft. RB, TBN, PN, OSB, CD and FMS reviewed and edited the manuscript. All authors participated in the interpretation of data and revising of the manuscript, and the final approval of the manuscript for submission.

## FUNDING

The Suubi+Adherence study was supported by funding from the Eunice Kennedy Shriver National Institutes of Child Health and Human Development (NICHD), grant number 1R01HD074949–01 (PI: Fred M. Ssewamala).

## DISCLAIMER

The contents of this manuscript are solely the responsibility of the authors and do not necessarily represent the official views of NICHD. The funders had no role in any of the stages of preparing this manuscript.

## Data Availability

The datasets included in the analysis for this study are available from the corresponding author on reasonable request.
